# Potential Application Value of FASN in the Diagnosis of Colorectal Cancer

**DOI:** 10.1002/jgh3.70261

**Published:** 2025-09-22

**Authors:** Nan Li, Mingyue Hu, Qiliu Qian, Jun Ouyang, Yulin Yang, Yongqi Zhang

**Affiliations:** ^1^ Department of Gastroenterology Zhongda Hospital, Southeast University Nanjing China; ^2^ Zhenjiang Hospital Affiliated to Nanjing University of Chinese Medicine Zhenjiang China; ^3^ Zhenjiang Traditional Chinese Medicine Spleen and Stomach Disease Clinical Medicine Research Center Zhenjiang China; ^4^ Department of Gastroenterology Xuyi People's Hospital Huaian China

**Keywords:** colorectal cancer, diagnosis, fatty acid synthase, prognosis

## Abstract

**Background:**

Fatty acid synthase (FASN) is a crucial enzyme that catalyzes endogenous lipogenesis in multiple diseases. However, the function and significance of FASN in colorectal cancer remain unclear.

**Aims:**

This research aimed to explore the expression and role of FASN in colorectal cancer.

**Results:**

Cancer, adjacent, and normal tissues were collected from patients with colorectal cancer and healthy controls. Immunohistochemistry and scoring were applied to analyze the expression of FASN in the different tissues, to investigate the differences in its expression among different tissue types, and to examine the potential correlation between FASN and gender, age, BMI, and other factors.

**Discussion:**

In this study, 100 colorectal cancer patients and 100 healthy participants were recruited. Respectively, the average scores of cancer tissues, adjacent tissues, and normal tissues were 7.25, 2, and 1.25. Significant differences were found among these three tissue groups (*p* < 0.05). Moreover, no significant association was observed between sex, age, BMI, and FASN expression scores in colorectal cancer tissues (*p* > 0.05).

**Conclusion:**

Based on these data, FASN was specifically overexpressed in cancer tissues and adjacent tissues. Hence, our results suggest that FASN may play an essential role in colorectal cancer and may be an attractive therapeutic target in the future.

## 1 Introduction

According to changes of diet and sedentary lifestyle, colorectal cancer has become the third place of incidence and the second place of mortality in the world [1]. Standard oncologic surgical resection is the main treatment for colorectal cancer, but this therapy is limited by early metastasis and various complications. Hence, early detection, accurate diagnosis, and effective treatment are essential for improving outcomes, and a deeper understanding of its mechanism will provide new ideas.

Fatty acid synthase (FASN) is a crucial enzyme that plays a pivotal role in the synthesis of fatty acids in cells, that is, important lipid molecules in building organisms. Fatty acid synthesis is vital for maintaining cellular functions. In addition to serving as an energy storage form, fatty acids also play a critical role in the structure and function of cell membranes. Furthermore, fatty acids serve as precursors for the synthesis of other important biomolecules, including hormones and cell‐signaling molecules [2]. Fatty acid synthase is a key enzyme in the intracellular synthesis of fatty acids that facilitates the conversion of substrates into long‐chain fatty acids through a series of chemical reactions. Fatty acid synthase synthesizes long‐chain saturated or unsaturated fatty acids by progressively lengthening the carbon chains of substrates such as acetyl‐CoA and propionyl‐CoA. In this process, fatty acid synthase catalyzes the continuous reaction of multiple enzyme activities, including dehydratase, cyclase, and reductase, generating different structures and saturations of fatty acids in each reaction step.

The activity and expression levels of FASN in cells can be influenced by a variety of regulatory mechanisms, including hormones, nutritional status, and transcription factors. Elevated expression and activity of FASN are associated with the development and progression of a number of diseases, including obesity, type II diabetes, and tumors. Consequently, FASN has been identified as a potential drug target that can regulate the development of certain diseases by inhibiting their activity.

Based on previous studies, we aimed to assess the expression of FASN in colorectal cancer tissues, adjacent tissues, and healthy tissues by immunohistochemistry. We also discuss the effects of FASN in patients with colorectal cancer.

## 2 Methods

### 2.1 Study Design and Patients

One hundred patients with colorectal cancer and 100 healthy participants were recruited from Zhongda Hospital Affiliated to Southeast University in 2022. Cancer tissues and adjacent tissues were obtained from colorectal cancer patients, and normal tissues were obtained from healthy controls for further immunohistochemical analysis; the analysis target was the FASN gene. All volunteers signed an informed consent document, and the project was approved by the Ethics Committee of Zhongda Hospital affiliated to Southeast University.

### 2.2 Immunohistochemistry and Scoring

The tissue microarrays were stained using the EnVision System. The primary antibody used was FASN Rabbit monoclonal antibody (#3180; Cell Signaling Technology, Danvers, MA, USA). FASN was determined by the presence of a brown reaction product in the cytoplasm of cells. Staining was evaluated by two pathologists, and the intensity of positive expression was scored using the coverage and cumulative intensity scores. The coverage score was measured according to the percentage of positively stained cells: 0, ≤ 5% stained; 1, 6%–25% stained; 2, 26%–50% stained; 3, 51%–75% stained; and 4, > 75% stained. The intensity score was measured as follows: 0, colorless; 1, weak; 2, moderate; and 3, intense. Then, the percentage and intensity scores were multiplied to produce a weighted score for each specimen: 0, negative (−); 1–4, weak positive (+); 5–8, positive (++); 9–12, intense positive (+++). The final score for each specimen was determined using the mean value of the two final scores.

### 2.3 Statistical Analysis

Data were statistically analyzed using IBM SPSS Statistics for Windows version 22.0. Normality tests were performed using the Shapiro–Wilk test and are presented as median (M) and four digits (P25, P75) if the data did not follow a normal distribution. The Kolmogorov–Smirnov *Z* test was used to compare two independent groups of samples, and the Kruskal–Wallis H test was used to compare multiple groups of samples. Univariate analysis was performed using analysis of variance, and regression analysis was performed using multiple linear regression *p* < 0.05. Multiple linear regression was used to analyze the factors influencing adjacent tissue scores.

## 3 Results

### 3.1 FASN Was Significantly Strongly Expressed in Cancer Tissues and Adjacent Tissues

Compared to normal tissues, FASN expression in cancer tissues and adjacent non‐cancerous tissues was markedly stronger. As shown in Table 1, cancer tissues scored on average 7.25 (*P*_25_, *P*_75_ 5.00, 10.50), which was higher than adjacent tissues scored on average 2 (*P*_25_, *P*_75_ 1.00, 3.50), as well as normal tissues scored on average 1.25 (*P*_25_, *P*_75_ 0.50, 1.50). Moreover, significant differences in the FASN expression scores were found among the three groups (*p* < 0.05).

**TABLE 1:** Comparison of tissue scores between groups [M (*P*_25_, *P*_75_)].

Group	Score	*H*	*p*
Cancer tissues (*n* = 100)	7.25 (5.00, 10.50)^a^	153.39	0.00
Paracancerous tissues (*n* = 100)	2 (1.00, 3.50)^a^		
Normal tissues (*n* = 100)	1.25 (0.50, 1.50)		

^a^ *p* < 0.05 compared with normal group.

After univariate analysis of different influencing factors and FASN expression scores, no significant association was observed between sex, age, BMI, and FASN expression scores in colorectal cancer tissue (Table 2, *p* > 0.05).

**TABLE 2:** Univariate analysis of different influencing factors.

	Sample size	Score	*F/t*	*p*
Gender			−0.78	0.44
Male	60	7.48 ± 3.27		
Female	40	8.01 ± 3.51		
Age			1.61	0.11
< 60	46	8.27 ± 3.25		
≥ 60	54	7.19 ± 3.40		
BMI (Kg/m^2^)			13.8	0.25
Thinnish	12	9.21 ± 2.58		
Normal	64	3.44 ± 0.43		
Overweight	1	12		
A little fat	15	3.33 ± 0.86		
Obesity	8	3.45 ± 1.22		
Tumor size (cm)			0.28	0.78
< 4.5	44	7.80 ± 3.19		
≥ 4.5	56	7.61 ± 3.51		
Tumor site			−0.71	0.48
Rectum	57	7.48 ± 3.02		
Colon	43	7.97 ± 3.78		
TNM			0.19	0.83
I	8	8.25 ± 3.21		
II	50	7.52 ± 3.36		
III	42	7.79 ± 3.46		
Lymph node metastasis			0.41	0.68
Yes	43	7.85 ± 3.44		
No	57	7.57 ± 3.33		
Tumor tissue type			2.31	0.11
Adenocarcinoma	84	7.87 ± 3.19		
Mucinous adenocarcinoma	15	6.30 ± 3.97		
Signet‐ring cell carcinoma	1	12		
Pathological differentiation of tumor			0.30	0.88
Highly differentiated	2	10.25 ± 0.35		
High‐medium differentiation	7	7.50 ± 2.33		
Moderately differentiated	73	7.66 ± 3.39		
Medium‐low differentiation	12	7.71 ± 3.83		
Poorly differentiated	6	7.42 ± 4.00		

## 4 Discussion

In this study, we investigated the differences in FASN expression among cancer, adjacent, and normal tissues and explored the possible influencing factors. According to the data, we found there were significant differences in FASN expression among these three groups. Moreover, this study also explored the factors affecting the scores of adjacent tissues by univariate analysis of 100 samples and found no significant association between factors such as sex, age, BMI index, and the scores of adjacent tissues.

FASN is an important enzyme that catalyzes the reaction between acetyl‐CoA and propionyl‐CoA to synthesize long‐chain unsaturated fatty acids. Normally, it is expressed at minimal levels in normal tissues, but at aberrant levels in a multitude of tumor types. Dysregulated fatty acid synthesis is regarded as an important therapeutic target in colorectal cancer, and FASN functions as an enzyme for de novo synthesis. Consistent with previous studies, our data showed that FASN is overexpressed in colorectal cancer tissues [3, 4]. Elevated FASN expression is related to poor clinical outcomes [5, 6]. This suggests that FASN may play an essential role in sustaining CRC development and progression in colorectal malignancy.

In this study, our results showed that there was no significant association between the FASN expression score and factors such as gender, age, BMI, and other factors. The underlying mechanisms of how FASN promotes colorectal cancer development have not yet been investigated. In addition to regulating fatty acid metabolism, FASN can maintain glycolysis and the tricarboxylic acid cycle in colorectal cancer [7]. Moreover, FASN also enhances oxaliplatin resistance, which is partly due to the MAPK/ERK and PI3K/AKT pathway [5]. In summary, targeting FASN may be a therapeutic option for colorectal treatment.

In conclusion, this study comparatively evaluated the expression of FASN in colorectal, adjacent, and normal tissues. Moreover, the correlation between FASN expression and several interfering factors was analyzed. However, further studies are needed to explore the underlying mechanisms of FASN in colorectal cancer development and progression.

## Ethics Statement

This research proposal was reviewed and approved by the ethics committee of Zhongda Hospital affiliated with Southeast University (2023ZDSYLL100‐P01).

## Consent

Written informed consent for publication was obtained from all the participants.

## Conflicts of Interest

The authors declare no conflicts of interest.

## Data Availability

Data are provided in the manuscript or in supplementary information files. Please contact Dr. Yongqi Zhang (e‐mail address: 13852315029@163.com) for questions or other requests regarding the study data and results.
